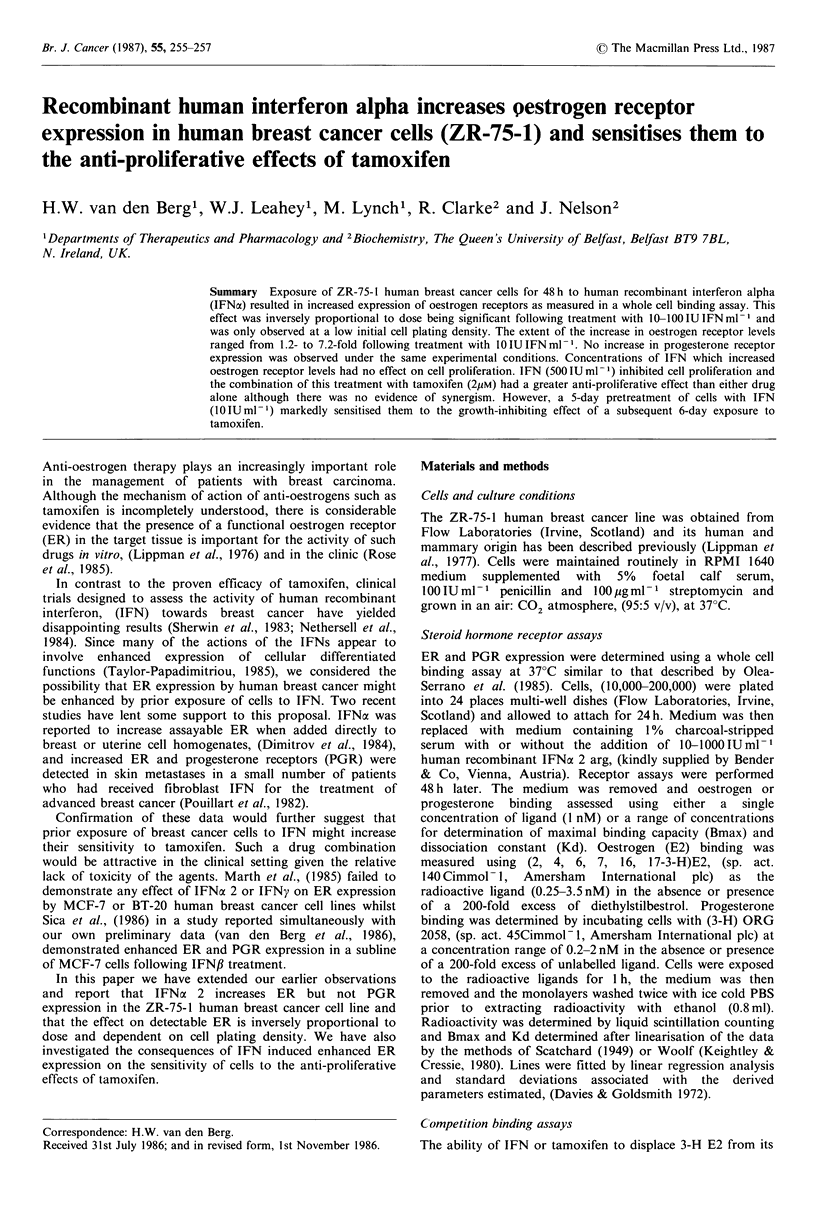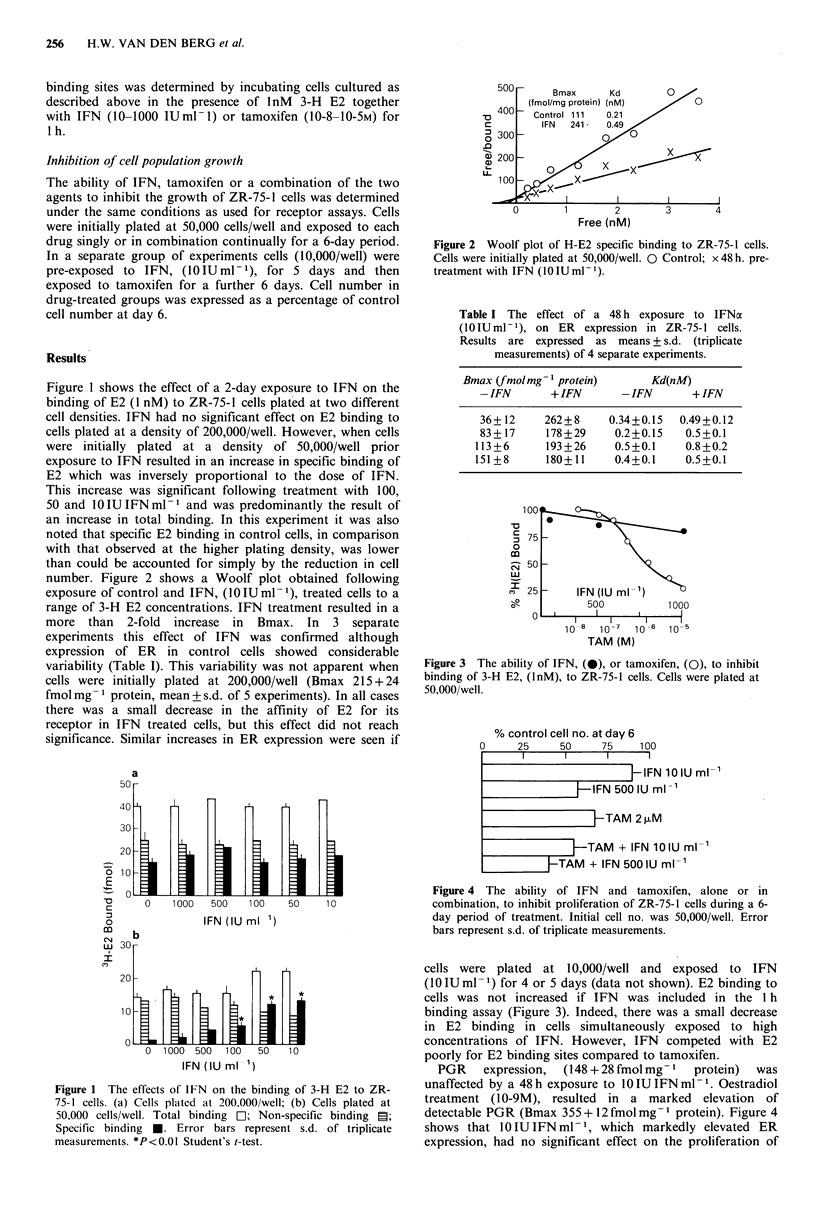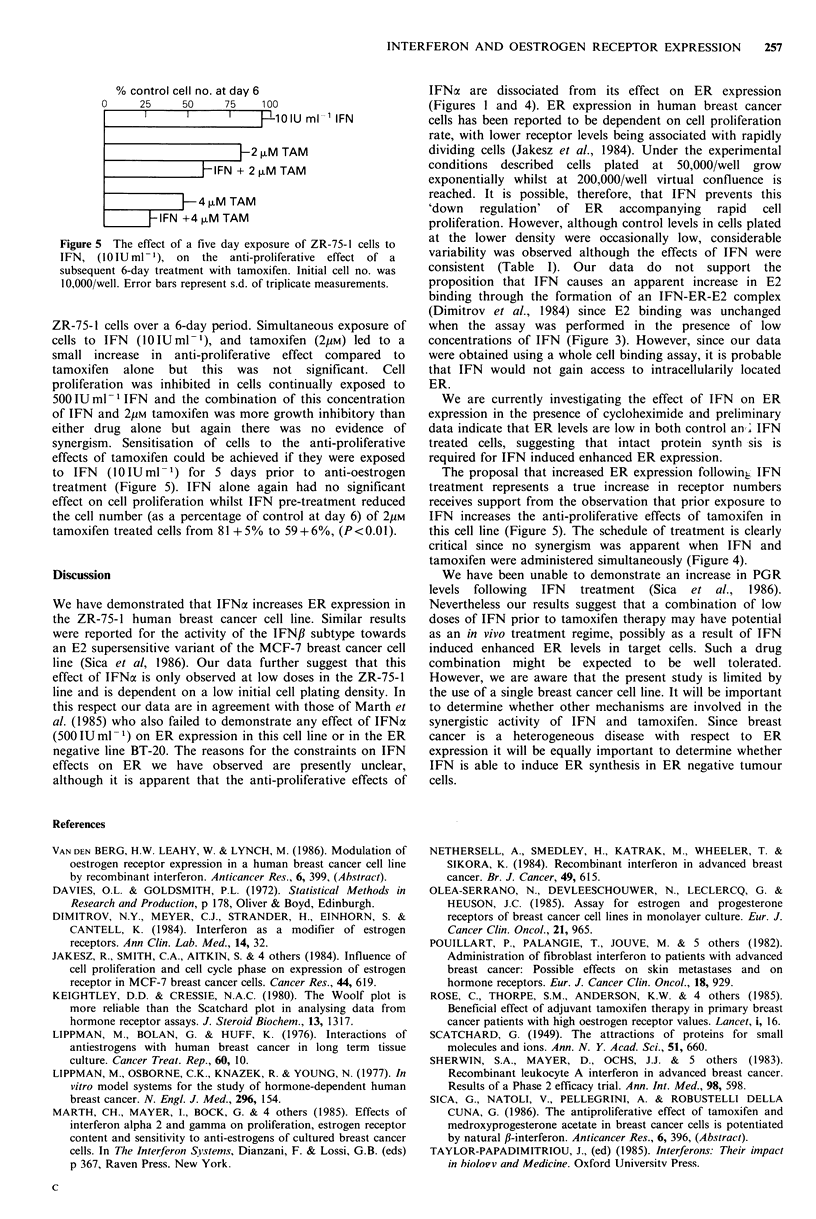# Recombinant human interferon alpha increases oestrogen receptor expression in human breast cancer cells (ZR-75-1) and sensitizes them to the anti-proliferative effects of tamoxifen.

**DOI:** 10.1038/bjc.1987.49

**Published:** 1987-03

**Authors:** H. W. van den Berg, W. J. Leahey, M. Lynch, R. Clarke, J. Nelson

## Abstract

Exposure of ZR-75-1 human breast cancer cells for 48 h to human recombinant interferon alpha (IFN alpha) resulted in increased expression of oestrogen receptors as measured in a whole cell binding assay. This effect was inversely proportional to dose being significant following treatment with 10-100 IU IFN ml-1 and was only observed at a low initial cell plating density. The extent of the increase in oestrogen receptor levels ranged from 1.2- to 7.2-fold following treatment with 10 IU IFN ml-1. No increase in progesterone receptor expression was observed under the same experimental conditions. Concentrations of IFN which increased oestrogen receptor levels had no effect on cell proliferation. IFN (500 IU ml-1) inhibited cell proliferation and the combination of this treatment with tamoxifen (2 microM) had a greater anti-proliferative effect than either drug alone although there was no evidence of synergism. However, a 5-day pretreatment of cells with IFN (10 IU ml-1) markedly sensitised them to the growth-inhibiting effect of a subsequent 6-day exposure to tamoxifen.


*Br. J. Cancer* (1987), **55**, 255–257 © The Macmillan Press Ltd., 1987

### Recombinant human interferon alpha increases oestrogen receptor expression in human breast cancer cells (ZR-75-1) and sensitises them to the anti-proliferative effects of tamoxifen

H.W. van den Berg[1], W.J. Leahey[1], M. Lynch[1], R. Clarke[2] and J. Nelson[2]

[1]*Departments of Therapeutics and Pharmacology and* [2]*Biochemistry, The Queen's University of Belfast, Belfast BT9 7BL, N. Ireland, UK.*

**Summary** Exposure of ZR-75-1 human breast cancer cells for 48 h to human recombinant interferon alpha (IFNα) resulted in increased expression of oestrogen receptors as measured in a whole cell binding assay. This effect was inversely proportional to dose being significant following treatment with 10–100 IU IFN $ml^{-1}$ and was only observed at a low initial cell plating density. The extent of the increase in oestrogen receptor levels ranged from 1.2- to 7.2-fold following treatment with 10 IU IFN $ml^{-1}$. No increase in progesterone receptor expression was observed under the same experimental conditions. Concentrations of IFN which increased oestrogen receptor levels had no effect on cell proliferation. IFN (500 IU $ml^{-1}$) inhibited cell proliferation and the combination of this treatment with tamoxifen (2 μM) had a greater anti-proliferative effect than either drug alone although there was no evidence of synergism. However, a 5-day pretreatment of cells with IFN (10 IU $ml^{-1}$) markedly sensitised them to the growth-inhibiting effect of a subsequent 6-day exposure to tamoxifen.

Anti-oestrogen therapy plays an increasingly important role in the management of patients with breast carcinoma. Although the mechanism of action of anti-oestrogens such as tamoxifen is incompletely understood, there is considerable evidence that the presence of a functional oestrogen receptor (ER) in the target tissue is important for the activity of such drugs *in vitro*, (Lippman *et al.*, 1976) and in the clinic (Rose *et al.*, 1985).

In contrast to the proven efficacy of tamoxifen, clinical trials designed to assess the activity of human recombinant interferon, (IFN) towards breast cancer have yielded disappointing results (Sherwin *et al.*, 1983; Nethersell *et al.*, 1984). Since many of the actions of the IFNs appear to involve enhanced expression of cellular differentiated functions (Taylor-Papadimitriou, 1985), we considered the possibility that ER expression by human breast cancer might be enhanced by prior exposure of cells to IFN. Two recent studies have lent some support to this proposal. IFNα was reported to increase assayable ER when added directly to breast or uterine cell homogenates, (Dimitrov *et al.*, 1984), and increased ER and progesterone receptors (PGR) were detected in skin metastases in a small number of patients who had received fibroblast IFN for the treatment of advanced breast cancer (Pouillart *et al.*, 1982).

Confirmation of these data would further suggest that prior exposure of breast cancer cells to IFN might increase their sensitivity to tamoxifen. Such a drug combination would be attractive in the clinical setting given the relative lack of toxicity of the agents. Marth *et al.*, (1985) failed to demonstrate any effect of IFNα 2 or IFNγ on ER expression by MCF-7 or BT-20 human breast cancer cell lines whilst Sica *et al.*, (1986) in a study reported simultaneously with our own preliminary data (van den Berg *et al.*, 1986), demonstrated enhanced ER and PGR expression in a subline of MCF-7 cells following IFNβ treatment.

In this paper we have extended our earlier observations and report that IFNα 2 increases ER but not PGR expression in the ZR-75-1 human breast cancer cell line and that the effect on detectable ER is inversely proportional to dose and dependent on cell plating density. We have also investigated the consequences of IFN induced enhanced ER expression on the sensitivity of cells to the anti-proliferative effects of tamoxifen.

Correspondence: H.W. van den Berg.
Received 31st July 1986; and in revised form, 1st November 1986.

### Materials and methods

### *Cells and culture conditions*

The ZR-75-1 human breast cancer line was obtained from Flow Laboratories (Irvine, Scotland) and its human and mammary origin has been described previously (Lippman *et al.*, 1977). Cells were maintained routinely in RPMI 1640 medium supplemented with 5% foetal calf serum, 100 IU $ml^{-1}$ penicillin and 100 μg $ml^{-1}$ streptomycin and grown in an air: $CO_2$ atmosphere, (95:5 v/v), at 37°C.

### *Steroid hormone receptor assays*

ER and PGR expression were determined using a whole cell binding assay at 37°C similar to that described by Olea-Serrano *et al.* (1985). Cells, (10,000–200,000) were plated into 24 places multi-well dishes (Flow Laboratories, Irvine, Scotland) and allowed to attach for 24 h. Medium was then replaced with medium containing 1% charcoal-stripped serum with or without the addition of 10–1000 IU $ml^{-1}$ human recombinant IFNα 2 arg, (kindly supplied by Bender & Co, Vienna, Austria). Receptor assays were performed 48 h later. The medium was removed and oestrogen or progesterone binding assessed using either a single concentration of ligand (1 nM) or a range of concentrations for determination of maximal binding capacity (Bmax) and dissociation constant (Kd). Oestrogen (E2) binding was measured using (2, 4, 6, 7, 16, 17-3-H)E2, (sp. act. 140 Cimmol$^{-}$1, Amersham International plc) as the radioactive ligand (0.25–3.5 nM) in the absence or presence of a 200-fold excess of diethylstilbestrol. Progesterone binding was determined by incubating cells with (3-H) ORG 2058, (sp. act. 45Cimmol$^{-}$1, Amersham International plc) at a concentration range of 0.2–2 nM in the absence or presence of a 200-fold excess of unlabelled ligand. Cells were exposed to the radioactive ligands for 1 h, the medium was then removed and the monolayers washed twice with ice cold PBS prior to extracting radioactivity with ethanol (0.8 ml). Radioactivity was determined by liquid scintillation counting and Bmax and Kd determined after linearisation of the data by the methods of Scatchard (1949) or Woolf (Keightley & Cressie, 1980). Lines were fitted by linear regression analysis and standard deviations associated with the derived parameters estimated, (Davies & Goldsmith 1972).

### *Competition binding assays*

The ability of IFN or tamoxifen to displace 3-H E2 from its binding sites was determined by incubating cells cultured as described above in the presence of lnM 3-H E2 together with IFN (10–1000 IU ml⁻1) or tamoxifen (10-8–10-5M) for 1 h.

*Inhibition of cell population growth*

The ability of IFN, tamoxifen or a combination of the two agents to inhibit the growth of ZR-75-1 cells was determined under the same conditions as used for receptor assays. Cells were initially plated at 50,000 cells/well and exposed to each drug singly or in combination continually for a 6-day period. In a separate group of experiments cells (10,000/well) were pre-exposed to IFN, (10 IU $ml^{-1}$), for 5 days and then exposed to tamoxifen for a further 6 days. Cell number in drug-treated groups was expressed as a percentage of control cell number at day 6.

### Results

Figure 1 shows the effect of a 2-day exposure to IFN on the binding of E2 (1 nM) to ZR-75-1 cells plated at two different cell densities. IFN had no significant effect on E2 binding to cells plated at a density of 200,000/well. However, when cells were initially plated at a density of 50,000/well prior exposure to IFN resulted in an increase in specific binding of E2 which was inversely proportional to the dose of IFN. This increase was significant following treatment with 100, 50 and 10 IU IFN $ml^{-1}$ and was predominantly the result of an increase in total binding. In this experiment it was also noted that specific E2 binding in control cells, in comparison with that observed at the higher plating density, was lower than could be accounted for simply by the reduction in cell number. Figure 2 shows a Woolf plot obtained following exposure of control and IFN, (10 IU $ml^{-1}$), treated cells to a range of 3-H E2 concentrations. IFN treatment resulted in a more than 2-fold increase in Bmax. In 3 separate experiments this effect of IFN was confirmed although expression of ER in control cells showed considerable variability (Table I). This variability was not apparent when cells were initially plated at 200,000/well (Bmax 215+24 fmol $mg^{-1}$ protein, mean ± s.d. of 5 experiments). In all cases there was a small decrease in the affinity of E2 for its receptor in IFN treated cells, but this effect did not reach significance. Similar increases in ER expression were seen if

**Figure 1** The effects of IFN on the binding of 3-H E2 to ZR-75-1 cells. (a) Cells plated at 200,000/well; (b) Cells plated at 50,000 cells/well. Total binding □; Non-specific binding ▤; Specific binding ■. Error bars represent s.d. of triplicate measurements. *$P<0.01$ Student's *t*-test.

**Figure 2** Woolf plot of H-E2 specific binding to ZR-75-1 cells. Cells were initially plated at 50,000/well. ○ Control; × 48 h. pre-treatment with IFN (10 IU $ml^{-1}$).

**Table I** The effect of a 48 h exposure to IFNα (10 IU $ml^{-1}$), on ER expression in ZR-75-1 cells. Results are expressed as means ± s.d. (triplicate measurements) of 4 separate experiments.

| *Bmax (fmol mg⁻¹ protein)* | | *Kd(nM)* | |
|---|---|---|---|
| *−IFN* | *+IFN* | *−IFN* | *+IFN* |
| 36±12 | 262±8 | 0.34±0.15 | 0.49±0.12 |
| 83±17 | 178±29 | 0.2±0.15 | 0.5±0.1 |
| 113±6 | 193±26 | 0.5±0.1 | 0.8±0.2 |
| 151±8 | 180±11 | 0.4±0.1 | 0.5±0.1 |

**Figure 3** The ability of IFN, (●), or tamoxifen, (○), to inhibit binding of 3-H E2, (lnM), to ZR-75-1 cells. Cells were plated at 50,000/well.

**Figure 4** The ability of IFN and tamoxifen, alone or in combination, to inhibit proliferation of ZR-75-1 cells during a 6-day period of treatment. Initial cell no. was 50,000/well. Error bars represent s.d. of triplicate measurements.

cells were plated at 10,000/well and exposed to IFN (10 IU $ml^{-1}$) for 4 or 5 days (data not shown). E2 binding to cells was not increased if IFN was included in the 1 h binding assay (Figure 3). Indeed, there was a small decrease in E2 binding in cells simultaneously exposed to high concentrations of IFN. However, IFN competed with E2 poorly for E2 binding sites compared to tamoxifen.

PGR expression, (148 + 28 fmol $mg^{-1}$ protein) was unaffected by a 48 h exposure to 10 IU IFN $ml^{-1}$. Oestradiol treatment (10-9M), resulted in a marked elevation of detectable PGR (Bmax 355 + 12 fmol $mg^{-1}$ protein). Figure 4 shows that 10 IU IFN $ml^{-1}$, which markedly elevated ER expression, had no significant effect on the proliferation of

**Figure 5** The effect of a five day exposure of ZR-75-1 cells to IFN, ($10\,IU\,ml^{-1}$), on the anti-proliferative effect of a subsequent 6-day treatment with tamoxifen. Initial cell no. was 10,000/well. Error bars represent s.d. of triplicate measurements.

ZR-75-1 cells over a 6-day period. Simultaneous exposure of cells to IFN ($10\,IU\,ml^{-1}$), and tamoxifen ($2\mu M$) led to a small increase in anti-proliferative effect compared to tamoxifen alone but this was not significant. Cell proliferation was inhibited in cells continually exposed to $500\,IU\,ml^{-1}$ IFN and the combination of this concentration of IFN and $2\mu M$ tamoxifen was more growth inhibitory than either drug alone but again there was no evidence of synergism. Sensitisation of cells to the anti-proliferative effects of tamoxifen could be achieved if they were exposed to IFN ($10\,IU\,ml^{-1}$) for 5 days prior to anti-oestrogen treatment (Figure 5). IFN alone again had no significant effect on cell proliferation whilst IFN pre-treatment reduced the cell number (as a percentage of control at day 6) of $2\mu M$ tamoxifen treated cells from $81+5\%$ to $59+6\%$, ($P<0.01$).

### Discussion

We have demonstrated that IFNα increases ER expression in the ZR-75-1 human breast cancer cell line. Similar results were reported for the activity of the IFNβ subtype towards an E2 supersensitive variant of the MCF-7 breast cancer cell line (Sica *et al*, 1986). Our data further suggest that this effect of IFNα is only observed at low doses in the ZR-75-1 line and is dependent on a low initial cell plating density. In this respect our data are in agreement with those of Marth *et al.* (1985) who also failed to demonstrate any effect of IFNα ($500\,IU\,ml^{-1}$) on ER expression in this cell line or in the ER negative line BT-20. The reasons for the constraints on IFN effects on ER we have observed are presently unclear, although it is apparent that the anti-proliferative effects of IFNα are dissociated from its effect on ER expression (Figures 1 and 4). ER expression in human breast cancer cells has been reported to be dependent on cell proliferation rate, with lower receptor levels being associated with rapidly dividing cells (Jakesz *et al.*, 1984). Under the experimental conditions described cells plated at 50,000/well grow exponentially whilst at 200,000/well virtual confluence is reached. It is possible, therefore, that IFN prevents this 'down regulation' of ER accompanying rapid cell proliferation. However, although control levels in cells plated at the lower density were occasionally low, considerable variability was observed although the effects of IFN were consistent (Table I). Our data do not support the proposition that IFN causes an apparent increase in E2 binding through the formation of an IFN-ER-E2 complex (Dimitrov *et al.*, 1984) since E2 binding was unchanged when the assay was performed in the presence of low concentrations of IFN (Figure 3). However, since our data were obtained using a whole cell binding assay, it is probable that IFN would not gain access to intracellularily located ER.

We are currently investigating the effect of IFN on ER expression in the presence of cycloheximide and preliminary data indicate that ER levels are low in both control and IFN treated cells, suggesting that intact protein synthesis is required for IFN induced enhanced ER expression.

The proposal that increased ER expression following IFN treatment represents a true increase in receptor numbers receives support from the observation that prior exposure to IFN increases the anti-proliferative effects of tamoxifen in this cell line (Figure 5). The schedule of treatment is clearly critical since no synergism was apparent when IFN and tamoxifen were administered simultaneously (Figure 4).

We have been unable to demonstrate an increase in PGR levels following IFN treatment (Sica *et al.*, 1986). Nevertheless our results suggest that a combination of low doses of IFN prior to tamoxifen therapy may have potential as an *in vivo* treatment regime, possibly as a result of IFN induced enhanced ER levels in target cells. Such a drug combination might be expected to be well tolerated. However, we are aware that the present study is limited by the use of a single breast cancer cell line. It will be important to determine whether other mechanisms are involved in the synergistic activity of IFN and tamoxifen. Since breast cancer is a heterogeneous disease with respect to ER expression it will be equally important to determine whether IFN is able to induce ER synthesis in ER negative tumour cells.

### References

VAN DEN BERG, H.W. LEAHY, W. & LYNCH, M. (1986). Modulation of oestrogen receptor expression in a human breast cancer cell line by recombinant interferon. *Anticancer Res.*, **6**, 399, (*Abstract*).

DAVIES, O.L. & GOLDSMITH, P.L. (1972). *Statistical Methods in Research and Production*, p 178, Oliver & Boyd, Edinburgh.

DIMITROV, N.Y., MEYER, C.J., STRANDER, H., EINHORN, S. & CANTELL, K. (1984). Interferon as a modifier of estrogen receptors. *Ann Clin. Lab. Med.*, **14**, 32.

JAKESZ, R., SMITH, C.A., AITKIN, S. & 4 others (1984). Influence of cell proliferation and cell cycle phase on expression of estrogen receptor in MCF-7 breast cancer cells. *Cancer Res.*, **44**, 619.

KEIGHTLEY, D.D. & CRESSIE, N.A.C. (1980). The Woolf plot is more reliable than the Scatchard plot in analysing data from hormone receptor assays. *J. Steroid Biochem.*, **13**, 1317.

LIPPMAN, M., BOLAN, G. & HUFF, K. (1976). Interactions of antiestrogens with human breast cancer in long term tissue culture. *Cancer Treat. Rep.*, **60**, 10.

LIPPMAN, M., OSBORNE, C.K., KNAZEK, R. & YOUNG, N. (1977). *In vitro* model systems for the study of hormone-dependent human breast cancer. *N. Engl. J. Med.*, **296**, 154.

MARTH, CH., MAYER, I., BOCK, G. & 4 others (1985). Effects of interferon alpha 2 and gamma on proliferation, estrogen receptor content and sensitivity to anti-estrogens of cultured breast cancer cells. In *The Interferon Systems*, Dianzani, F. & Lossi, G.B. (eds) p 367, Raven Press. New York.

NETHERSELL, A., SMEDLEY, H., KATRAK, M., WHEELER, T. & SIKORA, K. (1984). Recombinant interferon in advanced breast cancer. *Br. J. Cancer*, **49**, 615.

OLEA-SERRANO, N., DEVLEESCHOUWER, N., LECLERCQ, G. & HEUSON, J.C. (1985). Assay for estrogen and progesterone receptors of breast cancer cell lines in monolayer culture. *Eur. J. Cancer Clin. Oncol.*, **21**, 965.

POUILLART, P., PALANGIE, T., JOUVE, M. & 5 others (1982). Administration of fibroblast interferon to patients with advanced breast cancer: Possible effects on skin metastases and on hormone receptors. *Eur. J. Cancer Clin. Oncol.*, **18**, 929.

ROSE, C., THORPE, S.M., ANDERSON, K.W. & 4 others (1985). Beneficial effect of adjuvant tamoxifen therapy in primary breast cancer patients with high oestrogen receptor values. *Lancet*, **i**, 16.

SCATCHARD, G. (1949). The attractions of proteins for small molecules and ions. *Ann. N. Y. Acad. Sci.*, **51**, 660.

SHERWIN, S.A., MAYER, D., OCHS, J.J. & 5 others (1983). Recombinant leukocyte A interferon in advanced breast cancer. Results of a Phase 2 efficacy trial. *Ann. Int. Med.*, **98**, 598.

SICA, G., NATOLI, V., PELLEGRINI, A. & ROBUSTELLI DELLA CUNA, G. (1986). The antiproliferative effect of tamoxifen and medroxyprogesterone acetate in breast cancer cells is potentiated by natural β-interferon. *Anticancer Res.*, **6**, 396, (*Abstract*).

TAYLOR-PAPADIMITRIOU, J., (ed) (1985). *Interferons: Their impact in biology and Medicine*. Oxford University Press.